# Three-Dimensional (3D) Vertical Resistive Random-Access Memory (VRRAM) Synapses for Neural Network Systems

**DOI:** 10.3390/ma12203451

**Published:** 2019-10-22

**Authors:** Wookyung Sun, Sujin Choi, Bokyung Kim, Junhee Park

**Affiliations:** 1 Department of Electronic and Electrical Engineering, Ewha Womans University, Seoul 03760, Korea; sujinchoi26@gmail.com (S.C.); bkkim0505@hanmail.net (B.K.); 2 Medical Research Institute, Ewha Womans University, Seoul 03760, Korea

**Keywords:** RRAM, vertical RRAM, neuromorphics, neural network hardware, reinforcement learning

## Abstract

Memristor devices are generally suitable for incorporation in neuromorphic systems as synapses because they can be integrated into crossbar array circuits with high area efficiency. In the case of a two-dimensional (2D) crossbar array, however, the size of the array is proportional to the neural network’s depth and the number of its input and output nodes. This means that a 2D crossbar array is not suitable for a deep neural network. On the other hand, synapses that use a memristor with a 3D structure are suitable for implementing a neuromorphic chip for a multi-layered neural network. In this study, we propose a new optimization method for machine learning weight changes that considers the structural characteristics of a 3D vertical resistive random-access memory (VRRAM) structure for the first time. The newly proposed synapse operating principle of the 3D VRRAM structure can simplify the complexity of a neuron circuit. This study investigates the operating principle of 3D VRRAM synapses with comb-shaped word lines and demonstrates that the proposed 3D VRRAM structure will be a promising solution for a high-density neural network hardware system.

## 1. Introduction

In recent years, neuromorphic computing has emerged as a complementary system to the von Neumann architecture. Much of the research on neural network hardware implementation discusses how to connect large numbers of neurons and synapses. As a consequence, various memory devices such as static random-access memory, resistive random-access memory (RRAM), floating-gate (FG) memory, and phase change memory have been implemented as the synapse model in neural network hardware systems [1,2,3,4].

The most popular device-level component chosen to implement the synapses is the “memory resistor”, or memristor, because the resistance value of a memristor is a function of its historical activity. Moreover, energy efficiency is a key challenge of neuromorphic computing and RRAM is attractive for large-scale system demonstration due to its relatively lower energy consumption as compared with other synaptic devices [5]. The most common use of the memristor two-dimensional (2D)-crossbar is as a multiple memristor synapse since a single memristor cannot represent the positive and negative weights of synapses. However, 2D crossbar array synapses are not suitable for the implementation of deep neural networks (DNN) because the chip area depends on both the depth of the neural network and the number of input and output nodes.

**Figure 1:**
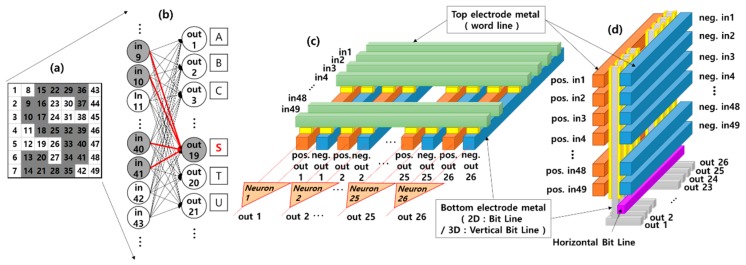
(**a**) The input pattern for letter ‘S’. (**b**) A neural network consisting of 49 input neurons and 26 output neurons (red lines = increased weights in the learning process) (**c**) Two-dimensional (2D) crossbar array synapses for implementing the neural network as shown in (b). (**d**) 3D vertical resistive random-access memory (VRRAM) synapses with the same performance as the synapses in (c).

The three-dimensional (3D) vertical resistance random-access memory (VRRAM) promises to minimize the area of a resistive memory. It can be categorized into two types based on its word line structures [6]: 3D VRRAM with a word line (WL) planar structure uses metal planes as WL electrodes, while a 3D VRRAM with a WL even/odd structure has comb-shaped WLs separated by etching. This structure is more promising than a WL plane structure for the VRRAM architecture because it has the same performance as a double cell bit [7,8]. Therefore, if a 3D VRRAM is used for synapses instead of a 2D crossbar array, as shown in Figure 1, the chip area of a DNN system can be effectively reduced. Recently, several works have evaluated the synaptic RRAM using 3D VRRAM. A high-density 3D synaptic architecture based on Ta/TaOx/TiO_2_/Ti RRAM is proposed as a neuromorphic computation hardware and the analog synaptic plasticity is simulated using the physical and compact models [9]. The potentiality of the VRRAM concept for various neuromorphic applications is investigated with one synapse being emulated by one VRRAM pillar [10]. Yet many of these studies have focused on experimental demonstration at a single RRAM cell level, and the idea that neuromorphic applications are possible is only presented as a concept. There are some previous studies related to 3D VRRAM with a WL planar structure. For example, the four-layer 3D RRAM integrated with FinFET (Fin Field-Effect Transistor) was developed for brain-inspired computing and in-memory computing [11], and 3D vertical array of RRAM was proposed for storing and computing large-scale weight matrices in the neural network [12]. However, a 3D VRRAM with comb-shaped WLs is more promising for a more efficient synaptic RRAM architecture because it has a double cell bit. Although research on 3D VRRAM with comb-shaped WLs has been published, it has focused on RRAM device variation, and explored the concept of many devices connected to one pillar operating as one synapse to overcome the variation [13]. Implementing a single synapse with multiple devices reduces the benefits of using 3D VRRAM. Moreover, reported previously related studies did not evaluate the circuit level properties of 3D VRRAM with comb-shaped WLs. Theoretical investigations are insufficient for exploring the relationship between synapse weight change and memory device resistance in 3D VRRAM.

In this study, we propose a new optimization method for machine learning weight changes that considers the structural characteristics of 3D VRRAM. This study investigates the operating principle of 3D VRRAM synapses with comb-shaped WLs and demonstrates that this structure is a promising synaptic model for neural network systems. The remainder of this paper is organized as follows: Section 2 describes a new 3D VRRAM crossbar array synapse incorporating a synaptic memristor model and learning operations for a guide training algorithm [14,15]. In Section 3, the accuracy of a neural network with 3D VRRAM synapses is measured by classifying 7 × 7 alphabet letter images using HSPICE circuit simulation. The conclusions are presented in Section 4.

## 2. Materials and Methods 

### 2.1. A Neural Network Learning Method Using a 3D VRRAM Synapse

**Figure 2:**
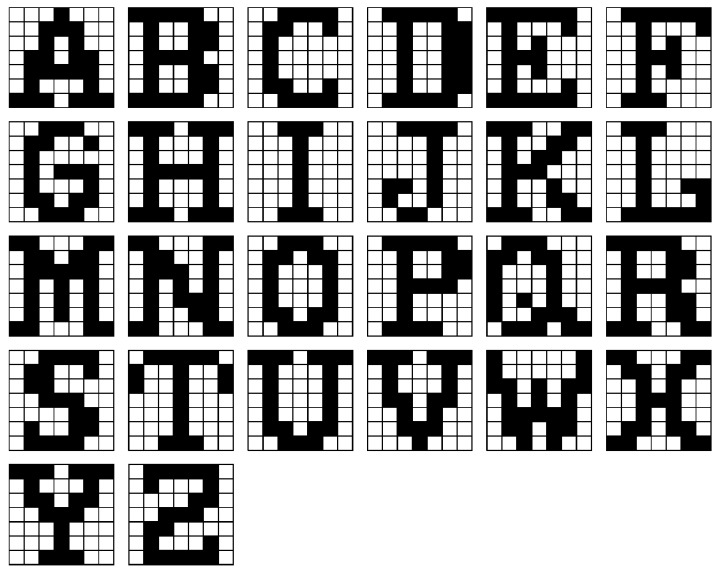
7 × 7 original alphabet images.

A neural network system design with 3D VRRAM synapses is shown in Figure 1. We evaluated the accuracy of the proposed 3D VRRAM synapses circuit by classifying 7 × 7 images representing alphabet letters as shown in Figure 2. Figure 1b shows a neural network consisting of 49 input neurons and 26 output neurons designed to classify input letter images into 26 classes as shown in Figure 1a. For the letter ‘S’, the nodes or neurons that generate the output spike are represented in gray, and increased weights in the learning process are indicated by red lines. The most common memristor application in neuromorphic systems is as the synapses in a 2D crossbar array as shown in Figure 1c. The weight of one synapse is represented by the conductance difference between two memristors because a single memristor cannot have both positive and negative weight values for a synapse [2]. For example, neuron 1 compares the total current of “positive out 1” in the red line with that of the “negative out 1” as shown in Figure 1c. If the “positive out 1” current is greater than the “negative out 1” current, neuron 1 spikes, which means the output of neuron 1 is a ‘1’. In contrast, when the “negative out 1” current is greater than the “positive out 1” current, the output of neuron 1 is ‘0’. The learning architecture for this implementation is constructed as a 49 × 52 2D memristor crossbar array.

If a 3D VRRAM is used for synapses, however, the chip area efficiency can be increased. Figure 1d shows a 3D VRRAM synapses structure with the same performance as Figure 1c. The ‘red’ and ‘blue’ word lines in Figure 1d represent “positive” and “negative” outputs, respectively. Therefore, only the area for 26 vertical pillars is needed to implement 26 classes in contrast to the need for 52 column lines in the 2D crossbar array. Moreover, the pillar structure of 3D VRRAM makes it simpler to build neuron circuits because there is no need for a circuit to a compare positive and negative current.

**Figure 3:**
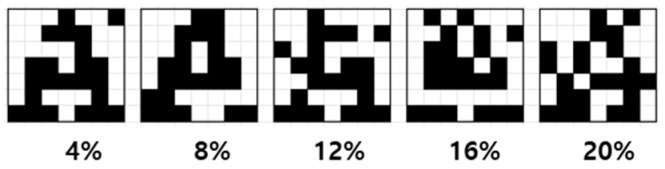
7 × 7 inverted pixel “A” image with noise from 4% to 20%.

A “guide training” algorithm is used to verify the accuracy and the performance of the 3D VRRAM synapses in HSPICE simulation [14,15]. This is a modified reinforcement learning algorithm and it is optimized for hardware implementation because it does not include a backpropagation algorithm. The algorithm was applied to image classification using the 2D crossbar memristor synaptic circuit, and its performance has been verified by showing a high learning success rate. The initial synaptic weights were randomized before the new training event was started. The single data set of 26 images (Figure 2), one for each alphabet letter, was defined as one epoch. After training, testing was performed to classify 20 test image sets consisting of the original or inverted pixel images, as shown in Figure 3. For example, the noise 0% test set consisted of 520 original images, and the noise 4% test set consisted of 520 images with two randomly selected pixels inverted.

### 2.2. 3D VRRAM Synapse Operation Mechanism

**Figure 4:**
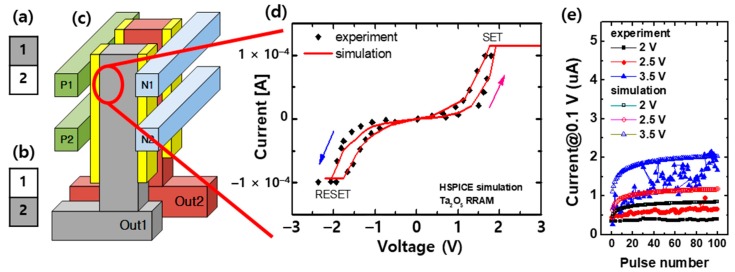
A two-pixel image where (**a**) pixel 1 is black and Out1 is “1”; (**b**) pixel 2 is black and Out2 is “1”; (**c**) 3D VRRAM synapse for a two-pixel image; (**d**) nonlinear I-V characteristic; and (**e**) linearly modulated potentiation behaviors of the Ta_2_O_5_ memristor device [21].

For this paper, we actually simulated the 3D VRRAM structure as shown in Figure 1d, but a description of the behavior of the real structure would be very complex. Therefore, we will explain the operation of 3D VRRAM with a simple structure as shown in Figure 4.

Figure 4a,b shows a simple two-pixel image to illustrate the weight change in a 3D VRRAM synapse configured as shown in Figure 4c. To categorize an image, a spike should be generated at the corresponding output or neuron of the input image. This means that a spike will occur at the Out1 neuron when Figure 4a is an input, and it will appear at the Out2 neuron if Figure 4b is an input. To allow a 3D VRRAM to operate as synapse circuit, its ‘Out1’ current must be larger than its ‘Out2’ current when Figure 4a is the input image. Conversely, if Figure 4b is an input image, Out2 current should be larger than Out1 current.

The 3D VRRAM in Figure 4c has a total of 8 memristors between its pillars (Out1 and Out2) and odd word lines (positive word line; P1, P2) or even word lines (negative word line; N1, N2). The number of word lines indicates the number of pixels. The memristor is a two-terminal device, so the “P1-Out1” memristor existing between the P1 word line and the Out1 pillar or vertical bit line is controlled by the bias of P1 and Out1. Reduced resistance in the memristors connected to the positive word line results in an increase in pillar current, while increased resistance of the memristor connected to the negative word line reduces the pillar current.

There are various memristor models for circuit simulation [16,17,18,19,20]. We used the generalized memristor model for this work [16,17], and it was coded in Verilog-A for the HSPICE circuit simulator. Figure 4d is the nonlinear I-V characteristic and Figure 4e is the linearly modulated potentiation behavior of an experimentally measured Ta_2_O_5_ memristor device [21]. It shows that the experiment and simulation results using our model are qualitatively consistent.

The memristor current is modeled by the hyperbolic sine function, as shown in Equation (1) [15,16]. Conductance is proportional to state variable x(t), which has a value between 0 and 1.

(1) $$I\left(t\right)=\left\{\begin{array}{c} a_{1}x\left(t\right)\sinh\left(bV\left(t\right)\right),\;\;V\left(t\right)\geq 0\\ a_{2}x\left(t\right)\sinh\left(bV\left(t\right)\right),\;\;V\left(t\right)<0 \end{array}\right\}$$

The change in the state variable over time is based on two different functions, g(V(t)) and f(x(t)).

(2) $$\frac{dx}{dt}=g\left(V\left(t\right)\right)f\left(x\left(t\right)\right)$$

(3) $$g\left(V\left(t\right)\right)=\left\{\begin{array}{c} A_{p}\left(e^{V\left(t\right)}-e^{V_{p}}\right),\;\;V\left(t\right)>V_{p}\\ -A_{n}\left(e^{-V\left(t\right)}-e^{V_{n}}\right),\;\;V\left(t\right)<-V_{n}\\ 0,\;\;-V_{n}\leq V\left(t\right)\leq V_{p} \end{array}\right\}$$

(4) $$f\left(x\left(t\right)\right)=\left\{\begin{array}{c} e^{-\alpha_{p}\left(x-x_{p}\right)}w_{p}\left(x,x_{p}\right),\;\;x\geq x_{p}\\ 1,\;\;\;\;\;\;\;\;\;\;\;\;\;\;\;\;x<x_{p} \end{array}\right\}$$

(5) $$f\left(x\left(t\right)\right)=\left\{\begin{array}{c} e^{\alpha_{n}\left(x+x_{n}-1\right)}w_{n}\left(x,x_{n}\right),\;\;x\leq 1-x_{n}\\ 1,\;\;\;\;\;\;\;\;\;\;\;\;\;\;\;\;\;\;x>1-x_{n} \end{array}\right\}$$

(6) $$w_{p}\left(x,x_{p}\right)=\frac{x_{p}-x}{1-x_{p}}+1$$

(7) $$w_{n}\left(x,x_{n}\right)=\frac{x}{1-x_{n}}$$

where g(V(t)) is a function of a programming threshold on the memristor model and f(x(t)) was used to limit the motion of the state variable (*x_p_* and *x_n_*). The function $w_{p}$ and $w_{n}$ are developed to limit the range of the state variable between 0 and 1. The model parameters used in this study are listed in Table 1.

**Figure 5:**
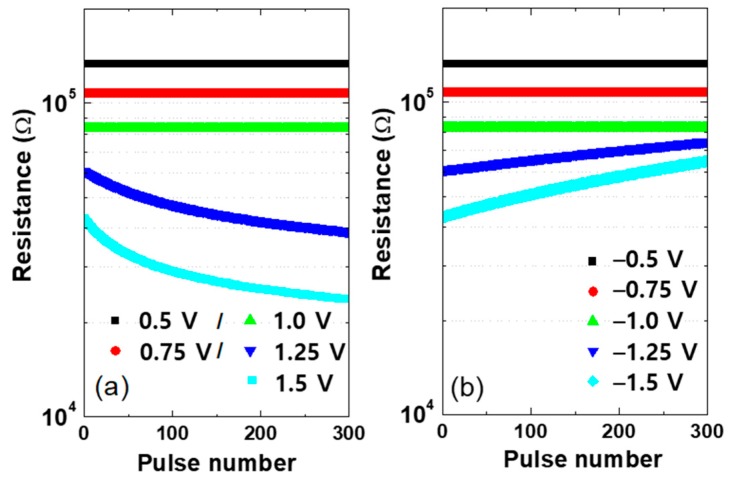
Resistance change of a memristor according to the (**a**) positive voltage and (**b**) negative voltage applied.

The memristor’s conductance changes from a high-resistance state (HRS) to a low-resistance state (LRS) when subjected to a voltage higher than the set voltage (= 1.2 V). A lower voltage than the reset voltage (= −1.2 V) changes the conductance of the memristor from an LRS to an HRS. The weight of a synapse or the resistance of each memristor could be changed during the network’s learning process but should be unchanged during the test process. To find the proper training voltage (Vtraining) and test voltage (Vtest), the change of resistance is simulated by applying various voltages to each memristor device. The voltage was applied from 0.5 V to 1.5 V or −0.5 V to −1.5 V at 0.25 V intervals. The unit pulse width is 10 ns and the rising and falling edge time is 0.5 ns. The line resistance of a vertical pillar is 3 Ω/cell with 20 nm class technology [8]. As shown in Figure 5a, the resistance changes only at 1.25 V and 1.5 V for five applied voltages because applying voltages greater than the set voltage (Vset) reduces resistance. Similarly, Figure 5b shows that the resistance changes at a voltage lower than the reset voltage (Vreset) but does not change at a higher voltage. Therefore, we set Vtraining = 1.5 V or −1.5 V, and Vtest = 1 V or −1 V considering the voltage drop in the crossbar array.

**Figure 6:**
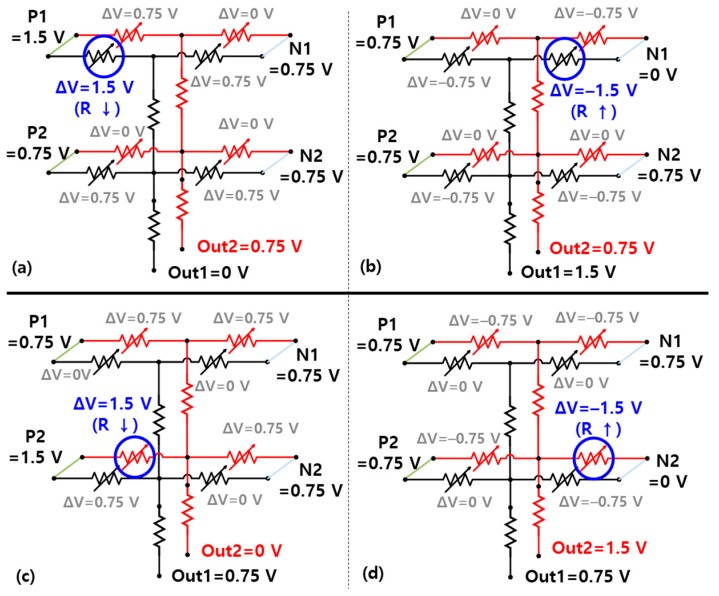
Simplified circuit diagrams of the 3D vertical synapse during the training procedure showing the voltages applied to (**a**) P1-Out1; (**b**) N1-Out1; (**c**) P2-Out2; and (**d**) N2-Out2 memristors when training Figure 4a,b.

First, the sequence of 3D VRRAM synapse learning is as follows. Figure 6 shows the circuit diagram of Figure 4c. If the input image is Figure 4a or Figure 4b, a spike is generated at the Out1 or Out2 neuron, respectively. In this study, we adopted the “winner-take-all” method to determine the neurons in which spikes occur. Thus, a spike in Out1 means that the current flowing to this neuron is the largest among the output neuron currents. Referring to Figure 6, the current of the Out1 neuron should be larger than the current of Out2 when the input image is Figure 4a. In the guide training method, only black pixel data is used for neural network learning, changing the weight of the synapse, or the resistance of the memristor connected to the black pixel [15].

Memristors connected to word lines P1 and P2 act as positive memristors that increase the weight of synapses. Increasing synaptic weights means that the resistance of the memristors is reduced, so Vtraining = 1.5 V is applied to P1 and P2 to increase the current flowing to Out1. In contrast, the memristor connected to N1 and N2 is a negative memristor that reduces the current in the Out1 neuron, and Vtraining = −1.5 V is applied to increase the resistance of the memristor. The number of positive and negative word line pairs matches the number of pixels. For example, P1 and N1 determine the characteristics of pixel 1 of the input image. 

An example of training for Figure 4a is illustrated in Figure 6a,b. The goal of the synapse learning is to lower the weight of synapses connected to black pixels, increasing the Out1 line current. In principle, all memristor devices connected to the Out1 line (P1-Out1, P2-Out1, N1-Out1, N2-Out1), which is shown in black lines in Figure 6, affect the generation of a spike when Figure 4a is the input image. However, since only pixel 1 is black in Figure 4a, the resistance of P1-Out1 and N1-Out1 is changed to increase the current of Out1 as shown in Figure 6a,b. In other words, the current of Out1 becomes larger than Out2 only when pixel 1 is black. Therefore, the resistance of “P1-Out1” should be reduced and that of “N1-Out1” should be increased to generate a spike on the Out1 neuron or increase Out1 current. 

The most important thing in the 3D vertical synapse learning process is that only the weights of the black pixel memristors change during learning, leaving other memristors unchanged. Therefore, to change the weight, a voltage greater than Vset is applied between the two electrodes of the P memristor, and a voltage less than Vreset is applied to its complementary N memristor. Figure 6a,b illustrates the training of the positive and negative memristors for the Figure 4a image and the Out1 neuron. During Out1 neuron training, Out2 remains at 0.75 V, and 0 V is applied to Out1 during positive memristor training and Vtraining (= 1.5 V) during negative memristor training. Basically, Vtraining (= 1.5 V) and 0 V are applied to the positive word line and negative word line, respectively, corresponding to the black pixels of the input image. The other four memristors (P1-Out2, P2-Out2, N1-Out2, N2-Out2), which are pictured with red lines in Figure 6, generate a spike on the Out2 neuron when the input is Figure 4b. Figure 6c,d shows the training procedure for Figure 4b like the training for the Out1 neuron.

**Figure 7:**
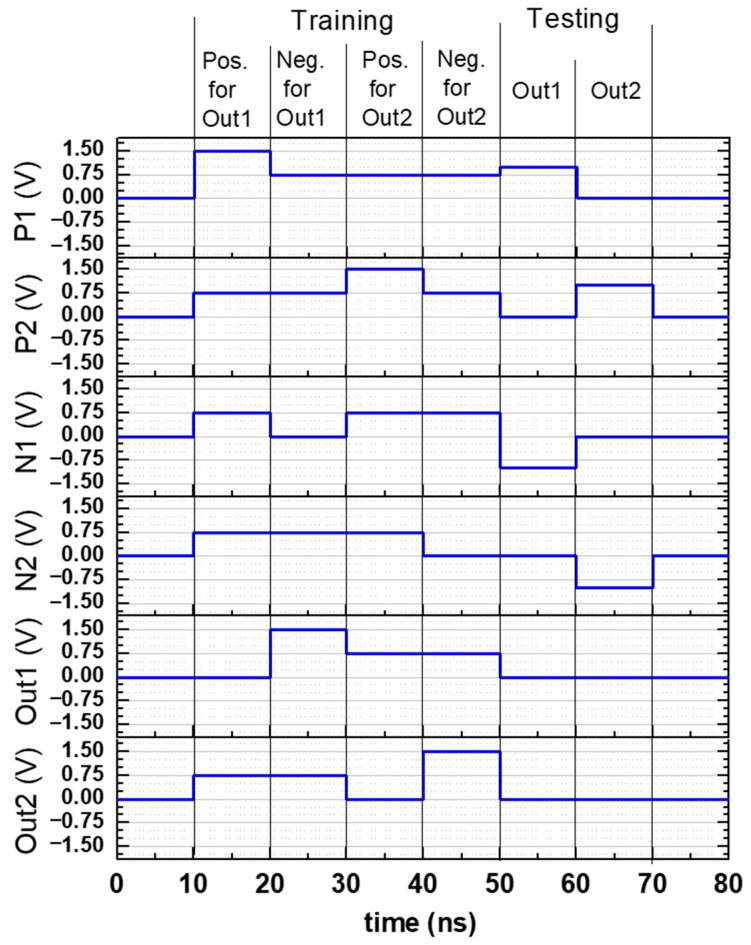
Input signal voltages at training and testing procedures.

The pillar of the 3D VRRAM connected to the Out1 neuron is used in common to train the positive and negative memristors. Therefore, the two processes should be done sequentially. The bias conditions for training and testing over time are shown in Figure 7. “Pos. for Out1” and “Neg. for Out1” represent the voltages that change the resistance of the positive and negative memristors. Since there are two pixels in Figure 4a,b, training occurs in a total of four sequences in Figure 7. The learning sequence increases in proportion to the number of pixels in the input image. The number of output neurons determines the number of test sequences. For example, if the input images are Figure 4a,b, we need two output sequences in this learning simulation.

**Figure 8:**
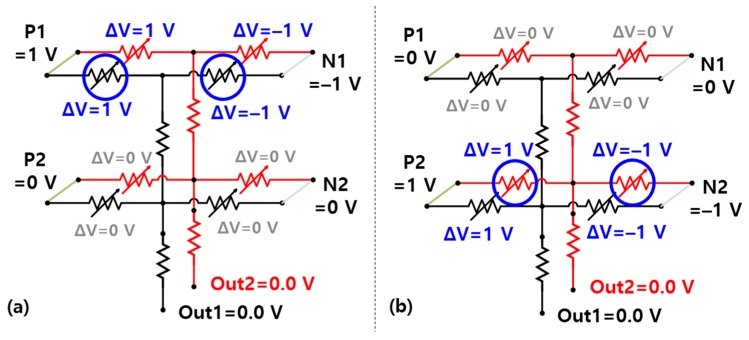
Simplified circuit diagrams of 3D vertical synapse during the testing procedure showing the test voltages applied to (**a**) the Out1 neuron and (**b**) the Out2 neuron.

Figure 8 shows the voltages in the simplified circuit diagrams of the 3D vertical synapses during the testing procedure. Unlike the learning process, the weight of the synapse (i.e., the resistance of the memristors), should not change during the testing process. Therefore, the test voltages are set to 1 V for the positive memristor and −1 V for the negative memristor, which are smaller than the set or reset voltages. During the learning process, the voltage applied to the memristor is determined by the difference between the voltage applied to the positive or negative word line and the voltage applied to the output line. During the test, however, the output line is held at 0 V and its current is determined only by the voltage applied to the word line. It means that 1 V and −1 V are respectively applied to the positive and negative word lines corresponding to black pixels. Therefore, when a voltage corresponding to Figure 4a is applied to the positive and negative word lines, the current of the Out1 neuron becomes larger than that of Out2 neuron, corresponding to the memristor resistances changed during the training process.

## 3. Results

To evaluate the accuracy of the proposed 3D VRRAM synapses, a guide training algorithm was tested by classifying the alphabet in 7 × 7 letter images in an HSPICE simulation. The initial synaptic weights were randomized before the start of the new training event. The single data set of 26 images (Figure 2), one for each alphabet letter, was defined as 1 epoch. After training, testing was performed to classify 20 test image sets consisting of the original or inverted pixel images. For example, the noise 0% test set consisted of 520 original images, and the noise 4% test set consisted of 520 images with two randomly selected pixels inverted.

**Figure 9:**
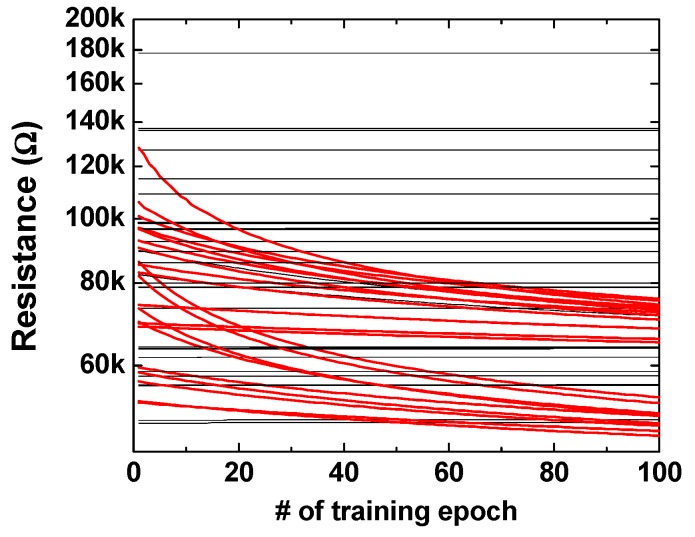
Resistance change of the positive memristors as a function of training epochs.

**Figure 10:**
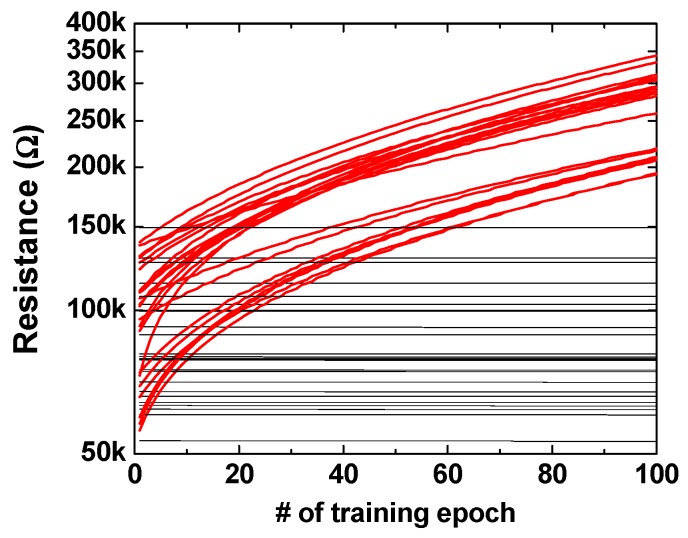
Resistance change of the negative memristors as a function of training epochs.

To confirm that the resistances were changed according to the training epoch, we applied the “S” image to the input and observed the synaptic change between the input neuron and the corresponding output neuron. Figure 9 shows the resistance change of the positive memristors according to the training epoch. There are 49 lines in the graph because the number of pixels or input neurons is 49. The training process enhances the synaptic weights of the input neurons associated with black pixels among the 49 pixels, and the enhancement of the synaptic weight means a decrease in resistance. The memristors with lowered resistance by training are shown by the red lines in Figure 9. In contrast to the positive memristors, the resistance of the negative memristors are increased by the training epoch. In Figure 10, as in Figure 9, only the memristors with increased resistance by training are shown in red.

## 4. Discussion

**Figure 11:**
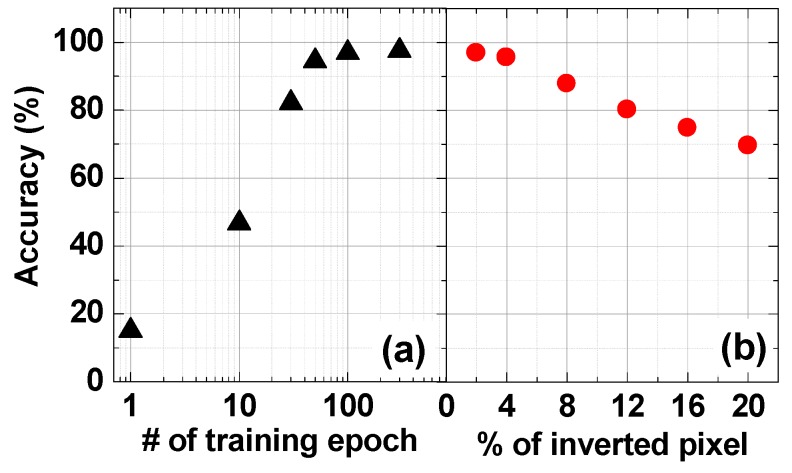
The accuracy of pattern classification after training according to (**a**) the number of training epochs and (**b**) the percentage of inverted pixels.

In order to determine the appropriate number of training epochs, the learning accuracy was evaluated by varying the number of training epochs from 1 to 300. Figure 11a shows the accuracy of pattern classification according to the number of training epochs. Only the original image was used in the test, and the accuracy of the pattern classification increases as the number of training epochs increases. The accuracy of the training after 100 epochs, however, is almost unchanged. Thus, we set 100 epochs as the default for neural network training simulation.

In order to verify how accurately the pattern classification can be performed even if noise is added to the input image, simulations were performed with an increasing number of inverted pixels as shown in Figure 11b. Obviously, as the noise increases in the input image, the accuracy of the pattern classification decreases. The simulation results, however, show 80% accuracy until the inverted pixel percentage increases to 12%. This means that 3D VRRAMs are usable as synapses in a neural network system. Therefore, using 3D VRRAM as the synapse structure of a neural network can greatly improve chip area utilization. In this study, we evaluated the accuracy of a neural network consisting only of input and output nodes with no hidden layers. A 3D VRRAM synapse with comb-shaped WLs structured with hidden layers is a subject for future work, and we will demonstrate the effects of 3D VRRAM synapses by performing simulations in a more diverse learning environment. 

## 5. Conclusions

In this study, a 3D VRRAM structure was newly proposed as the synapse of a neural network system. It was concluded that 3D VRRAM implemented as synapses can increase the chip area efficiency and simplify the neuron circuits. This study investigates the operating principle of 3D VRRAM using comb-shaped WL synapses and proves that this structure has promise for a neural network system. The accuracy of a neural network with 3D VRRAM synapses was measured by classifying 7 × 7 alphabet letter images using a circuit simulator. The guide training algorithm was optimized for hardware implementation because it does not include a backpropagation algorithm. Therefore, the guide training algorithm and winner-take-all methods were used to validate the performance accuracy of the 3D VRRAM synapses in a HSPICE simulation. The simulation results showed 80% accuracy until the inverted pixel count reached 12%. This means that 3D VRRAMs are usable as synaptic mimic circuits in neural network systems. A 3D vertical synapse with an integrated 3D VRRAM structure will be a promising solution for a high-density neuromorphic chip.

## Figures and Tables

**Table 1:** Parameters used in the synapse guide model.

Symbol	Value	Symbol	Value
a_1_	1 × 10^−5^	A_n_	1 × 10^7^
a_2_	1 × 10^−5^	x_p_	0.2
b	2.1	x_n_	0.25
V_p_	1 (V)	α_p_	7
V_n_	1 (V)	α_n_	6
A_p_	3 × 10^6^	x_o_	0.3
